# The Preparation of Glycerinated Calf Lymph

**Published:** 1898-05-28

**Authors:** 


### THE PREPARATION OF GLYCERINATED CALF LYMPH.

In the Milroy Lectures, Dr. Monckton Copeman gives full details of the mode of preparation of glycerinated calf lymph, the use of which it is proposed to make compulsory. The chief points are (1) the quarantining of the calf to ensure that it is healthy; (2) the sterilisation of the seat of inoculation; (3) the inoculation of the incisions, which are about four inches long and half an inch apart; (4) after five days the collection of the vaccine pulp. This is done by sterilised instruments amid as completely aseptic conditions as can be obtained. It is in this stage that the chief difficulty arises, the only organisms met with which resist the germicidal action of glycerine being the hay bacillus and the B. mesentericus, and their intrusion is doubtless due to difficulties in the way of cleansing the vesicles. In collecting the pulp the whole of the vesicles and their contents are removed by a Volkmann's spoon. (5) The preparation of the lymph pulp. This is first weighed, and is then thoroughly triturated, and when it has been brought to a state of fine division it is mixed with six times its weight of a sterilised solution of 50 per cent. pure glycerine in distilled water. The emulsion so produced is stored in test tubes in a cool, dark place, and when required is drawn up into sterilised capillary tubes, which are sealed as usual. (6) Bacteriological examination of the emulsion is made at regular intervals, and usually at the end of four weeks all extraneous organisms are found to have died out. (7) Meanwhile, after the collection of the pulp the calf is transferred to the slaughter-house and examined by the veterinary surgeon, who forwards a certificate of its condition. Should this not prove satisfactory the pulp is all destroyed. Looking at the matter from a scientific standpoint, no one can doubt the immense advance which this process shows in every point over the happy-go-lucky methods of vaccination which have been far to common in this country. We cannot but feel, however, that the manufacture of a product of this kind should be carried out under the strictest guarantees, such as can only be satisfactorily given by a Government institution; for there can be but little doubt that much of the prejudice which many medical men have felt against calf lymph has been due to the varying results which have been said to follow the use of samples derived from different sources.

